# Risk of self‐harm and suicide on reaching the age at which a parent died by suicide or other causes: A Danish, population‐based self‐controlled case series study

**DOI:** 10.1111/sltb.13135

**Published:** 2024-11-19

**Authors:** Yanakan Logeswaran, Keltie McDonald, Julie Cerel, Gemma Lewis, Annette Erlangsen, Alexandra Pitman

**Affiliations:** ^1^ UCL Division of Psychiatry London UK; ^2^ College of Social Work University of Kentucky Lexington Kentucky USA; ^3^ Psychiatric Centre Copenhagen Danish Research Institute for Suicide Prevention Copenhagen Denmark; ^4^ Copenhagen Research Centre for Mental Health, Mental Health Center Copenhagen Mental Health Services, Capital Region of Denmark Copenhagen Denmark; ^5^ Department of Mental Health Johns Hopkins School of Public Health Hampton Maryland USA; ^6^ Centre for Mental Health Research, Research School of Population Health The Australian National University Canberra Australia; ^7^ Camden and Islington NHS Foundation Trust St Pancras Hospital London UK

**Keywords:** age correspondence, attempted suicide, bereavement, grief, suicide, time factors

## Abstract

**Introduction:**

Risk factors for suicide after parental suicide may include points in the lifecourse when reminders of the deceased trigger grief resurgence. We hypothesized that risk of suicide attempt and suicide is elevated among suicide‐bereaved offspring on reaching the age at which a parent died by suicide.

**Methods:**

We conducted a self‐controlled case series (SCCS) study using national register data on all individuals bereaved by parental suicide living in Denmark from 1980 to 2016. We compared relative incidence of our combined outcome (any secondary‐care episode of self‐harm or suicide) during the exposure period (2 years centred on the birthday representing age correspondence) and the 15‐year unexposed periods either side. We repeated these models for offspring bereaved by parental non‐suicide death as an indirect comparison.

**Results:**

Risk of self‐harm or suicide was elevated on reaching the age at parental suicide (*n* = 188; IRR_adj_: 2.02, 95% CI: 1.21–3.38) relative to flanking periods, but not at parental non‐suicide death (*n* = 734; IRR_adj_: 0.76; 95% CI: 0.39–1.50).

**Conclusions:**

Reaching the age at which a parent died by suicide appears to represent a vulnerable period for suicidality, countering public perceptions that time heals linearly. This indicates a need for support in the lead up to age correspondence.

## INTRODUCTION

People bereaved by the suicide of a close friend, partner or relative have an increased risk of depression (Pitman et al., 2014), suicide attempt (Pitman et al., 2016) and suicide (Pitman et al., 2022). Most research has focussed on the impact of parental suicide, finding that suicide‐bereaved offspring have an almost three‐fold increased risk of suicide compared with offspring of living parents, and over double the risk for offspring bereaved by other causes of parental death (Calderaro et al., 2022). The mechanisms of the association between parental suicide bereavement and suicide are poorly understood, with likely genetic (Tidemalm et al., 2011), epigenetic (Calderaro et al., 2022) and environmental (Pitman et al., 2017) contributions. Putative environmental contributors include modeling of parental coping style, whilst post‐bereavement contributors include family disruption, loss of income, and grief of the surviving parent (Calderaro et al., 2022). Suicide‐bereaved offspring appear to be at greater suicide risk when the same gender as the deceased (Cheng et al., 2014), and are also likely to use similar methods (Ranning et al., 2022). Although genetic mechanisms for these gender differences are possible, other explanations include social learning of maladaptive responses to distress (Insel & Gould, 2008) and psychodynamic factors, such as identification with the deceased (Hung & Rabin, 2009). The relative contribution of these and genetic factors has not yet been clearly differentiated (Fazel & Runeson, 2020). It is important to investigate potential cognitive mechanisms underlying the association between suicide loss and suicide risk. Some may represent opportunities for reframing beliefs around suicide as inevitable and coping with grief, thereby reducing suicidal distress.

Cognitive factors might plausibly influence the timing of suicide risk among the suicide‐bereaved, for example around emotionally salient dates such as death anniversaries. The evidence for this is mixed, with Swedish population data indicating an elevated risk of suicide on death anniversaries in women bereaved by any cause (Grotta et al., 2023; Rostila et al., 2015) but a reduced risk in men bereaved by any cause (Grotta et al., 2023). Findings from Danish population data indicate no increased risk of suicide attempt or suicide around birthdays and anniversaries of the deceased, whether after suicide or after bereavement by other causes (Pitman et al., 2023). Other salient dates may have strong resonance for the bereaved, including the age correspondence on reaching the age of a deceased parent (Birtchnell, 1981; CALM, 2022). Clinical accounts suggest that such age correspondence may increase suicide risk (Birtchnell, 1981; Mintz, 1971), with possible contributors including reignition of unprocessed trauma (Chow, 2010) to the point of wishing for escape (Young et al., 2012), beliefs about inevitability (Pitman et al., 2017), yearning for reunion (Shear & Skritskaya, 2012; Young et al., 2012) and identification with the deceased (Birtchnell, 1981; Mintz, 1971). The relative contribution of each factor has not been investigated, but any resultant preoccupation with death at age correspondence might increase cognitive availability of suicide and of suicide method (Biddle et al., 2012; Buus Florentine & Crane, 2010), increasing acquired capability for suicide (O'Connor & Kirtley, 2018; Van Orden et al., 2010). Conversely, it is possible that reaching this age is experienced as a positive milestone, representing an important goal to surpass.

There is very limited research investigating these concepts in the context of suicide loss. Qualitative accounts from suicide‐bereaved relatives describe “death anxiety” relating to the heritability of psychiatric disorder or suicidality, and a fear that suicide may be inevitable (Pitman et al., 2017). Whether suicidal thoughts or behavior increase on approaching the age of a parent's suicide has, to our knowledge, not been investigated and could potentially identify opportunities for intervention. In this study we hypothesized that rates of self‐harm and suicide are higher on reaching the age attained by a parent who died by suicide when compared with flanking periods.

## METHODS

### Study design

We used a self‐controlled case series (SCCS) design using nationwide data on the entire Danish population. In a SCCS design, only individuals who were cases are included during both exposed and unexposed time periods (Whitaker et al., 2006). Individuals serve as their own controls; thus, by design, one is able to account for time‐fixed observed and unobserved confounders, including genetic or socio‐economic factors (Petersen et al., 2016), when comparing events during exposed and unexposed time periods.

### Data source

We analyzed linkage data on Danish‐born individuals who lived in Denmark at any point between January 1, 1980 and December 31, 2016, with no age restrictions. Using a unique personal identification number assigned to all individuals at birth (or first entry into the country) (Erlangsen & Fedyszyn, 2015), we linked individual‐level data from five nationwide population registers: the Civil Registration System (Pedersen, 2011), Register of Causes of Death (Helweg‐Larsen, 2011), Registry of Social Pension and Income (Baadsgaard & Quitzau, 2011), National Hospital Register (Andersen et al., 1999), and Psychiatric Central Research Register (Mors et al., 2011).

### Participants

As per the exposure and outcome definitions described below, our models only included participants who, during 1980–2016, experienced parental bereavement, reached an age that was 16 years prior to the birthday at which they attained the age of the deceased, had a recorded episode of self‐harm or suicide within the 16 years before or after that birthday, and did not migrate out of Denmark between bereavement and the first self‐harm/suicide event.

We identified all individuals who had been bereaved by parental death between January 1, 1980 and December 31, 2016. Relatives were identified using information on family type and household identification number, which are recorded in the Civil Registration System. Parents' deaths by suicide or other causes were identified using the relevant International Classification of Diseases (ICD)‐8 and ICD‐10 codes from the Register of Causes of Death (Supplementary Methods).

We created two separate samples for comparison: (a) individuals bereaved by parental suicide, and (b) individuals bereaved by parental death by other causes. Individuals who experienced more than one parental bereavement were included at the date of the first loss (index bereavement).

### Measures

#### Outcome

We used a combined outcome of all self‐harm episodes and suicide deaths recorded over each period of interest, allowing for recurrent events. Self‐harm was identified as psychiatric and somatic (secondary care) hospital contact for self‐harm in the Psychiatric Central Research Register and the National Patient Register based on relevant ICD‐8 and ICD‐10 codes or where the reason for contact was recorded as “self‐harm” (Supplementary Methods), as per the standard approach (Morthorst et al., 2016). We restricted self‐harm to episodes identified in secondary care in order to identify medically severe cases in which high suicidal intent was assumed. Suicides were identified from the Register of Causes of Death using relevant ICD‐8 and ICD‐10 codes (Supplementary Methods). Any self‐harm episode occurring within 7 days of a previously recorded self‐harm episode was considered as the same event, using the date of the first event. Similarly, any suicide occurring within 7 days of a previous self‐harm episode was considered as the same event, using the date of the suicide.

#### Exposure

**FIGURE 1:**
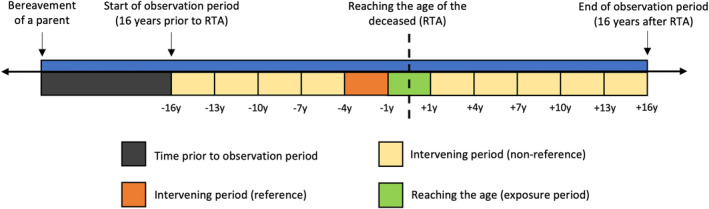
Representation of observation period for the examined SCCS models. The exposure period represents the 12 months either side of the birthday at which the bereaved attained the age of the deceased parent (two‐year interval in total). The unexposed period represents the 15‐year periods flanking the exposure period. The unexposed period is separated out into three‐year intervals, the last of which prior to the exposure (4 to <1 year prior) was chosen as the reference category.

The exposure was the 2‐year time period during which a bereaved individual reached the age attained by the deceased parent (index bereavement), operationalized as the 12 months prior to and after the birthday at which the bereaved attained that age (Figure 1). This was intended to capture anticipation of, attaining and surpassing that age. We defined unexposed time periods as the 15‐year periods before and after this 24‐month exposure time period, to correspond to the minimum intergenerational age difference of 16 years from beginning of the unexposed period to the salient birthday (for either sample).

#### Covariates

We adjusted for the following time‐varying covariates (defined in Supplementary Methods) based on existing evidence of possible confounding: age at start of each period categorized in 5‐year bands (Fazel & Runeson, 2020); household income level (Fazel & Runeson, 2020); and marital status (as distinct from cohabitation status, to capture the effect of divorce) (Kposowa, 2000). We opted not to adjust for hospital‐diagnosed depression, which might influence risk of self‐harm at an emotionally salient date, because recording of depression is influenced by a self‐harm event, thus violating an assumption of the SCCS approach (Petersen et al., 2016).

#### Follow‐up

We followed individuals from 15 years before the exposure period (i.e., 16 years before the birthday representing attaining the age of a deceased parent) until censorship due to own death by other causes, emigration, a second parental bereavement (if the second deceased parent was younger than the index deceased parent, as this would otherwise reset the exposure period to earlier), the end of the observation period (i.e., 16 years after the birthday representing attaining the age of a deceased parent) or December 31, 2016, whichever came first.

We used the adapted SCCS method for censored post‐event exposures, developed to accommodate fatal outcomes because a condition of the standard SCCS approach is that the outcome event should not censor subsequent observations (Farrington, 2022). Fatal outcomes (e.g., suicides) would normally censor subsequent observations, but the adapted SCCS method for censored post‐event exposures accommodates these by considering the theoretical risk of dying at any point during the study period (Farrington et al., 2009, 2011; Petersen et al., 2016; Whitaker et al., 2006). The adapted approach retains individuals within the model after a fatal outcome is recorded by extending follow‐up until the end of the intended observation period. An individual therefore contributes person‐days to the model after their death (but no events), continuing until the end of the observation period. This avoids truncating the person‐days at risk in the period during which they died relative to other periods, avoiding inflation of the event rate in that period relative to others.

As per the adapted SCCS design, following a suicide we retained individuals in the model so that they contributed person‐days at risk until the end of the observation period.

### Statistical analysis

Characteristics of each sample were assessed using descriptive statistics. Incident rates (total number of self‐harm episodes and suicide deaths in each period divided by the length of the period) were calculated, using fixed effects conditional Poisson regression to derive crude incidence rate ratios (IRRs) comparing exposed and unexposed periods, with corresponding 95% confidence intervals. As unexposed periods were up to 15 years long, these were subdivided into 3‐year intervals, the last of which prior to the exposure (4 to <1 year prior) was set as the reference category a priori, as per precedent (Wijlaars et al., 2013). We reported rates for each of the 3‐year intervals.

IRRs were adjusted for age, marital status, and household income level. Separate SCCS models were conducted for offspring bereaved by suicide and offspring bereaved by other causes, for indirect comparison.

In an a priori sensitivity analyses, we assessed for potential bias due to the inclusion of individuals experiencing a second parental bereavement where the parent was older than the first deceased parent. We did this by repeating our main models but censoring individuals after any second parental bereavement. To explore a methodological point and understand the effect of using more fine‐grained versus longer unexposed periods, we conducted an additional sensitivity analysis in which we grouped together (a) the intervening periods prior to reaching the age of the deceased and (b) the intervening after reaching the age of the deceased, using the earlier intervening period as a 15‐year reference category. We also conducted a post hoc sensitivity analysis in which we divided our 2‐year exposure period into the 12 months prior to, and 12 month after, the birthday at which the bereaved attained age correspondence, to report estimates for each of those subperiods.

Data preparation was performed in SAS software version 9.4 (SAS, 2003) and analyses were conducted in Stata 18 software (StataCorp., 2023).

### Ethical approval

The study was approved by the Danish Data Protection Agency and informed consent was waived. Institutional approval to analyze extracted data was provided by the University College London Research Ethics Committee (approval number 14075/001).

## RESULTS

### Sample characteristics

**FIGURE 2:**
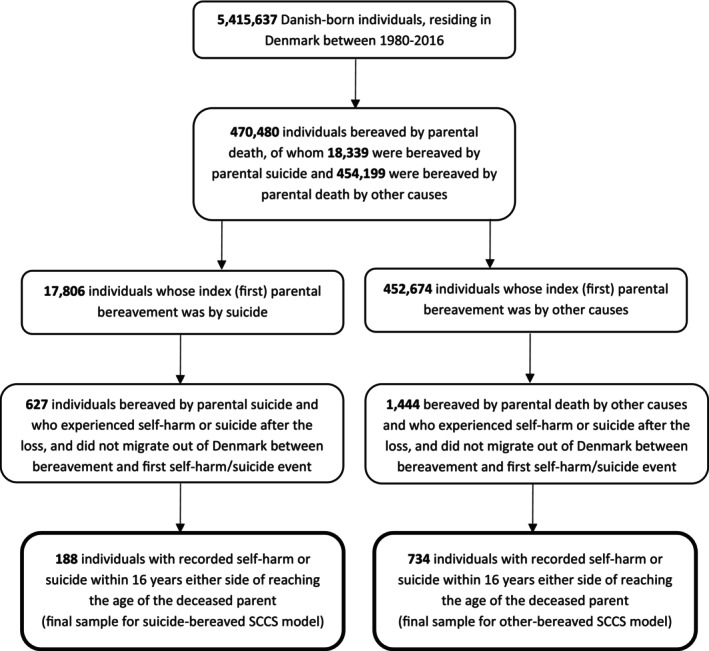
Flow of participants into SCCS models.

Of the 5,415,637 Danish‐born residents of Denmark at any point between 1980 and 2016, we identified 470,480 individuals bereaved by parental death over that period (Figure 2), of whom 18,339 were bereaved by parental suicide (17,806 as index bereavement) and 454,199 were bereaved by parental death by other causes (452,674 as index bereavement).

The median intergenerational age gap between offspring and parents for the Danish population as a whole over this period was 29.3 years (IQR = 25.5–33.4; range = 14.1–94.6), whilst for our SCCS sample of suicide‐bereaved offspring it was 23.7 years (IQR = 21.8–27.5; range = 17.6–37.9) and for our SCCS sample of other‐bereaved offspring it was 23.7 years (IQR = 21.0–27.2; range = 16.8–44.4).

### Suicide‐bereaved

Of the 17,806 people (0.3% of 5,415,637) who experienced index parental bereavement by suicide over this period, 627 were recorded with a subsequent self‐harm episode or suicide. Of these, 188 (30.0%) had the outcome of interest (158 self‐harm episodes; 46 suicides) within our defined observation period and were included as participants (Table 1). During the exposure period there were 27/188 (14.4%) with recorded outcomes.

**TABLE 1:** Characteristics of individuals in models for those bereaved by parental suicide and for those bereaved by other causes of parental death.

	Offspring bereaved by suicide (*n* = 188)		Offspring bereaved by other causes of death (*n* = 734)	
	Median	IQR	Median	IQR
Age in years^a^				
At bereavement	14	7–20	18	10–23
At start of follow‐up	22	15–28	26	17–32
At censorship	40.5	34–48	44	37–49
At first suicide attempt	29	21.5–35	33	25–41
Of deceased parent at death	38	31–45	42	34–49
Age difference between offspring and parent	23.7	21.8–27.5 (range = 17.6–37.9)	23.7	21.0–27.2 (range = 16.8–44.4)
	***n***	**%**	***n***	**%**
Sex				
Male	110	58.5	424	57.8
Female	78	41.5	310	42.2
Household income level^b^				
1 (lowest quartile)	46	24.5	161	21.9
2	54	28.7	216	29.4
3	53	28.2	227	30.9
4 (highest quartile)	31	16.5	118	16.1
Unknown	4	2.1	12	1.6
Marital status^b^				
Never married	174	92.6	662	90.2
Married/registered partnership	10	5.3	52	7.1
Divorced/dissolved partnership	<3	–	12	1.6
Widowed	0	0.0	<3	–
Unknown	3	1.6	7	1.0
Prior self‐harm (binary measure)^c^	9	4.8	31	4.2
	***n***	**IQR**	***n***	**IQR**
Number of self‐harm episodes during observational period	<3	1–1 (range = 1–13)	<3	1–1 (range = 1–21)
	***n***	**%**	***n***	**%**
Number of suicide deaths during observational period	46	24.5	222	30.3
Mental health disorders				
Any	8	4.3	41	5.6
PTSD	0	0.0	0	0.0
Depression	<3	–	<3	–
Anxiety	0	0.0	3	0.4
Substance use	6	3.2	34	4.6
Severe mental illness^d^	3	1.6	12	1.6
Physical health disorders				
Any	0	0.0	5	0.7
Cardiovascular disease	0	0.0	<3	–
Hypertension	0	0.0	<3	–
Diabetes mellitus	0	0.0	<3	–
COPD	0	0.0	<3	–

*Note*: Any figures quoted as <3 indicate that cell size was below the threshold for reporting exact figures, as per the Statistics Denmark stipulations on protecting confidentiality.

Abbreviations: COPD, chronic obstructive pulmonary disease; IQR, interquartile range; PTSD, post‐traumatic stress disorder.

^a^ Time‐varying covariate, using age of participant at start of each period.

^b^ Time‐varying covariate, values presented here are for the year prior to start of follow‐up (i.e., 15 years before the 2 year exposure period).

^c^ Covering years in between index bereavement and the start of follow‐up.

^d^ Severe mental illness was defined as psychotic disorders, manic episode, bipolar affective disorder, and depression with psychotic symptoms (see Supplementary Methods).

### Other‐bereaved

Of the 452,674 people (8.4% of 5,415,637) who experienced index parental bereavement by other causes over this period, 1444 were recorded with a subsequent self‐harm episode or suicide. Of these, 734 (40.8%) had the outcome of interest (570 self‐harm episodes; 222 suicides) within our observation period and were included as participants (Table 1). During the exposure period there were 62/734 (8.5%) with recorded outcomes.

Indirectly comparing the two samples, individuals bereaved by suicide were younger (median age = 14 years; IQR = 7–20) than those bereaved by other causes (median age = 18 years; IQR = 10–23). Median age of parental death in our suicide‐bereaved sample was 38.9 years (IQR = 31.9–45.3; range = 21.2–64.7) compared with 42.8 years (IQR = 34.4–49.0; range = 19.8–67.5) in our other‐bereaved sample.

#### Risk of self‐harm and suicide upon reaching the age of the deceased

**FIGURE 3:**
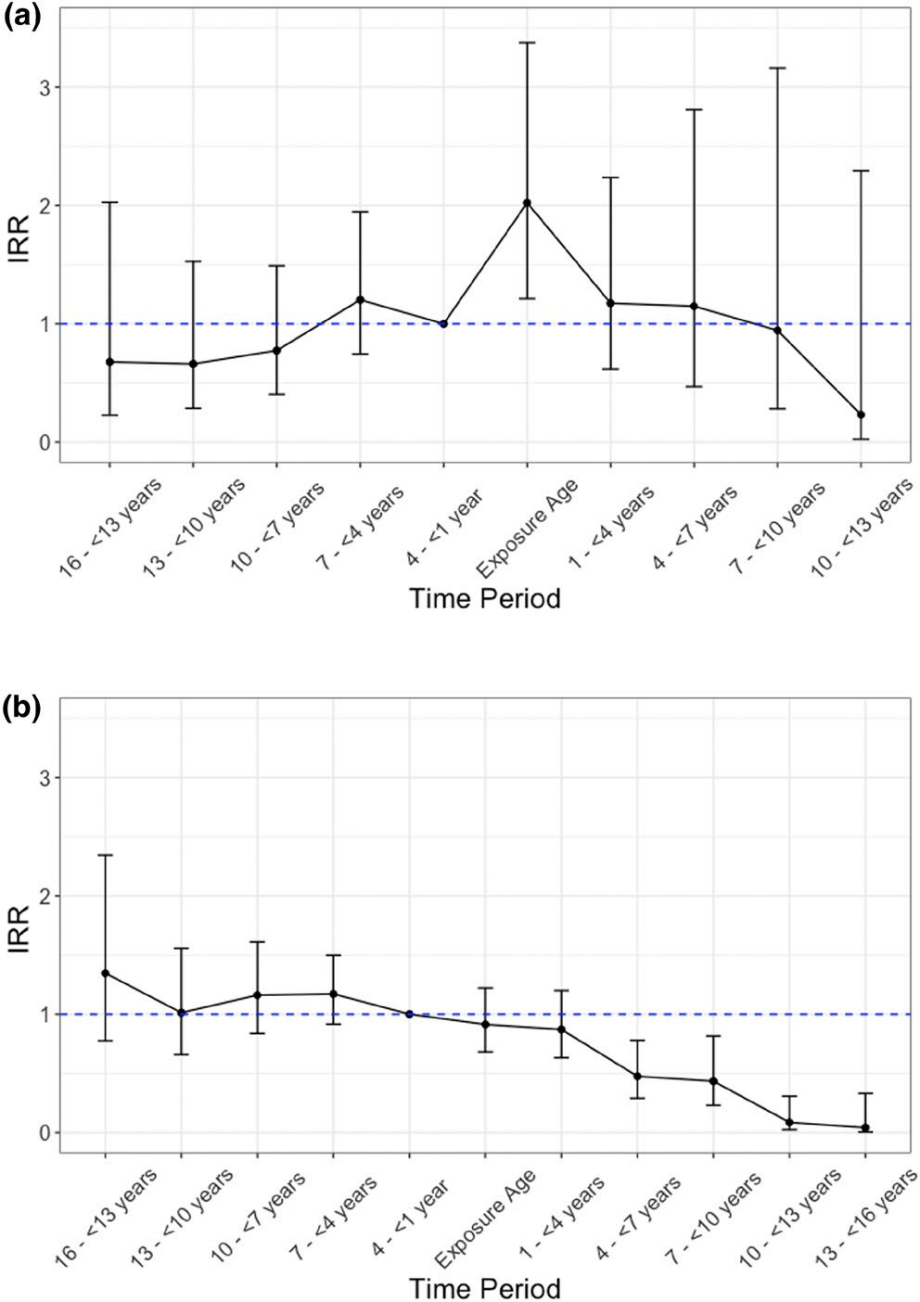
Graphical representation of risk of self‐harm or suicide by specific time periods over follow‐up in (a) suicide‐bereaved individuals and (b) other‐bereaved individuals (IRRs by period) in relation to attaining the age of the deceased parent. (a) Risk of suicide attempt and suicide on attaining the age (exposure period) at which a parent died by suicide. N=188 (1.03%) people were included in this model from a subsample of 18,339 individuals in Denmark who were bereaved by parental suicide and who were potentially eligible for the analysis. (b) Risk of suicide attempt and suicide on attaining the age (exposure period) at which a parent died by non‐suicide causes. N=734 (0.16%) people were included in this model from a subsample of 452,674 individuals in Denmark who were bereaved by parental non‐suicide death and who were potentially eligible for the analysis.

Offspring bereaved by parental suicide had an increased rate of self‐harm or suicide on attaining the age of the deceased parent (IRR_crude_: 1.65; 95% CI: 1.05–2.60; IRR_adj_: 2.02, 95% CI: 1.21–3.38; Table 2 and Figure 3) when compared with the reference period.

**TABLE 2:** Risk of suicide attempt by specific time periods over follow‐up in (a) suicide‐bereaved individuals and (b) other‐bereaved individuals (IRRs by period).

	Event	PDAR	Unadjusted IRR (95% CI)	*p*‐value	Adjusted^a^ (95% CI)	*p*‐value
Suicide‐bereaved						
Years prior to exposed time period						
16 to <13 years	53	206,180	1.47 (0.96–2.25)	0.074	0.68 (0.23–2.03)	0.486
13 to <10 years	45	206,014	1.25 (0.81–1.94)	0.318	0.66 (0.29–1.53)	0.332
10 to <7 years	41	206,004	1.14 (0.73–1.78)	0.569	0.77 (0.40–1.49)	0.444
7 to <4 years	53	205,994	1.47 (0.96–2.25)	0.073	1.20 (0.74–1.95)	0.451
4 to <1 year (reference period)	36	205,992	1.00 [ref.]	–	1.00 [ref.]	–
Exposed time period (Age of the deceased parent; 2‐year period)	39	134,821	1.65 (1.05–2.60)	0.030	2.02 (1.21–3.38)	0.007
After the age of the deceased parent						
1 to <4 years	26	170,202	0.85 (0.51–1.41)	0.533	1.17 (0.62–2.24)	0.626
4 to <7 years	15	131,867	0.64 (0.35–1.17)	0.144	1.15 (0.47–2.81)	0.761
7 to <10 years	7	98,409	0.40 (0.18–0.91)	0.028	0.94 (0.28–3.16)	0.925
10 to <13 years^b^	<3	<75,000	0.08 (0.01–0.56)	0.011	0.23 (0.02–2.29)	0.211
13 to <16 years^c^	0	57,639	–	0.985	–	0.976
Other‐bereaved						
Prior to the age of the deceased parent						
16 to <13 years	241	804,996	1.57 (1.29–1.93)	<0.001	1.35 (0.77–2.34)	0.291
13 to <10 years	177	804,290	1.16 (0.83–1.44)	0.187	1.01 (0.66–1.56)	0.955
10 to <7 years	196	804,287	1.28 (1.04–1.58)	0.022	1.16 (0.84–1.61)	0.370
7 to <4 years	190	804,283	1.24 (1.00–1.53)	0.046	1.17 (0.91–1.50)	0.210
4 to <1 year (reference period)	153	804,262	1.00 [ref.]	–	1.00 [ref.]	–
Age of the deceased parent (2‐year period)	85	524,615	0.85 (0.65–1.11)	0.237	0.91 (0.68–1.22)	0.538
After the age of the deceased parent						
1 to <4 years	95	647,235	0.79 (0.61–1.02)	0.071	0.87 (0.63–1.20)	0.398
4 to <7 years	34	496,355	0.39 (0.27–0.56)	<0.001	0.48 (0.29–0.78)	0.003
7 to <10 years	23	376,695	0.35 (0.23–0.55)	<0.001	0.43 (0.23–0.82)	0.010
10 to <13 years	3	305,532	0.06 (0.02–0.19)	<0.001	0.09 (0.02–0.31)	<0.001
13 to <16 years	<3	<265,000	0.02 (0.00–0.18)	<0.001	0.04 (0.01–0.33)	0.003

Abbreviations: CI, confidence interval; PDAR, person‐days at risk.

^a^ Adjusted for age, marital status, and household income level.

^b^ Figures quoted as <3 indicate that the cell size was below the threshold for reporting exact figures, as per the Statistics Denmark stipulations on protecting confidentiality. The corresponding PDAR is masked because of the possibility of identifying n through backcalculation of the PDAR.

^c^ There were no events in this period due to the low number of individuals who had been bereaved at least 29 years before the end of data collection and to have survived until this period. As this was not the reference period, this did not bias estimates.

Offspring bereaved by other causes of parental death did not have an increased rate of self‐harm or suicide on attaining the age of the deceased parent (IRR_crude_: 0.85; 95% CI: 0.65–1.11; IRR_adj_: 0.91; 95% CI: 0.68–1.22; Table 2 and Figure 3) when compared with the reference period.

### Sensitivity analyses

Similar to our main findings, an increased rate of self‐harm or suicide (IRR_crude_: 1.66; 95% CI: 1.04–2.64; IRR_adj_: 2.03, 95% CI: 1.20–3.43) was observed on attaining the age of a parent who died by suicide for a sample of suicide‐bereaved individuals who were censored at the point of any second parental bereavement (rather than solely where the second parental bereavement involved a younger parent) (Table S1). Again, as for our main findings, no difference was found between the rate of self‐harm or suicide (IRR_crude_: 0.90; 95% CI: 0.68–1.20; IRR_adj_: 0.98; 95% CI: 0.72–1.35) among offspring bereaved by other causes on attaining the age of a deceased parent when compared to the reference period.

Models using as reference category an aggregated period of 15 years prior to reaching the age were similar to our main findings. Among individuals bereaved by parental suicide, they showed an increased rate of self‐harm or suicide was observed on reaching the age of the deceased (IRR_crude_: 1.31; 95% CI: 0.93–1.83; IRR_adj_: 1.94, 95% CI: 1.26–2.96; Table S2). Again, individuals bereaved by other causes of parental death did not have an increased rate of self‐harm or suicide on attaining the age of the deceased parent (IRR_crude_: 0.68; 95% CI: 0.55–0.85; IRR_adj_: 1.03; 95% CI: 0.80–1.33; Table S2).

Results of our post hoc sensitivity analysis showed that, among offspring bereaved by suicide, an increased rate of self‐harm or suicide (IRR_crude_: 1.92, 95% CI: 1.14–3.23; IRR_adj_ = 2.38, 95% CI = 1.34–4.22) was observed in the year prior to age correspondence, but not in the year following (IRR_crude_: 1.38, 95% CI: 0.77–2.49; IRR_adj_ = 1.77, 95% CI = 0.90–3.45). Findings for offspring bereaved by other causes were similar to our main findings: no differences in rates of self‐harm or suicide were found in the year prior to age correspondence (IRR_crude_ = 0.98, 95% CI: 0.71–1.35; IRR_adj_ = 1.01, 95% CI = 0.72–1.42) or the year following (IRR_crude_: 0.72, 95% CI: 0.50–1.04; IRR_adj_ = 0.75, 95% CI = 0.50–1.11).

## DISCUSSION

### Main findings

We found that reaching the age at parental death was associated with an increased risk of medically serious self‐harm and suicide among suicide‐bereaved offspring but not offspring bereaved by other causes. This supports our theory that cognitive factors at this emotionally charged age may contribute to suicidal behavior after suicide, although we were unable to investigate mechanisms. We have theorized that such processes might include a rekindling of unprocessed trauma and grief, a desire to escape unbearable distress, beliefs about suicide being inevitable, yearning for reunion, and/or identification with the deceased, leading to a final common pathway of increased cognitive availability of suicide and acquired capability for suicide. However, these hypotheses require further investigation. Our post hoc sensitivity analysis suggests that risk is confined to the period approaching age correspondence, whilst acknowledging small numbers.

Despite death anxiety being observed in people bereaved by non‐suicide causes (Azaiza et al., 2011; Florian & Mikulincer, 1997), based on an understanding of the heritability of physical health problems such as cardiovascular disease, we did not observe an elevated self‐harm or suicide risk on reaching the age of a parent deceased by non‐suicide causes. This does not rule out increased needs for emotional support at such a stage, and any bereaved offspring may be vulnerable to anticipatory anxiety on approaching the age of a parent's death (Kouremetis, 2019).

Contemporary models of grief acknowledge that individuals move backwards and forwards between phases or stages of grief rather than in a linear manner (Bristowe et al., 2024). For example, in the dual process model of grief, the bereaved are conceived as oscillating between loss orientation, in which they focus on their grief and pain, and restoration orientation, in which they distract themselves from grief in order to cope (Stroebe & Schut, 1999). Our findings support the idea of a dynamic process of grief, in so much as the elevated risk of suicidal behavior at age correspondence might also represent a period of loss orientation and increased distress. We could not, however, test this empirically as the registry‐based dataset lacked variables recording severity of depressive or other symptoms. We can therefore only speculate as to the relationship of our findings to theories of grief. It is important to note that age correspondence occurred a median of 24 years after the loss. Support is unlikely to be readily available at this point given the perceptions of suicide‐bereaved people that others expect them to “move on” from their grief soon after the loss (Pitman, De Souza, et al., 2018).

Other factors may explain the elevated risk observed at age correspondence, including genetic influences on the age at which suicidality emerges in the offspring of suicide decedents and on the lethality of methods used (Ranning et al., 2022). Age‐related genetic penetrance describes the influence of age on the probability of suicidal behavior emerging among individuals with inherited risk for suicidal behavior. Whilst it is possible that suicidal behavior presents at a similar age in offspring of parents who die by suicide, modifiers of age at symptom onset are likely to include other sex‐linked genes and a range of environmental risk and protective factors. Such ideas remain theoretical and there is no clear evidence delineating the degree of genetic control over age at emergence of suicidal behavior (Fazel & Runeson, 2020). However, in our sample, when considering all suicidal behavior over the life course, the median age of first suicide attempt for suicide‐bereaved offspring (29 years) was 9 years before reaching the median age of parental death (38 years). As this does not occur at age correspondence, age‐related genetic penetrance is unlikely to be a dominant contributor to our findings regarding elevated risk of suicial behavior at age correspondence. It must also be acknowledged that suicide risk is generally elevated in mid‐life; a stage when suicide risk factors such as relationship breakdown, physical health problems, substance use, and unemployment accumulate (Shiner et al., 2009). Bereaved offspring (usually adolescents or young adults at the time of parental suicide) approaching the age of parental death will be influenced by similar sociodemographic and clinical risk factors as their parents (and their non‐bereaved peers). However, our models' restriction of the period of interest to the 15 years either side of age correspondence (in which mid‐life social risk factors might also apply) and our adjustment for age, income and marital status should account for these background factors.

### Findings in the context of other studies

To our knowledge, the hypothesis that suicide risk increases at emotionally salient dates relating to parental age at death has not previously been examined. Other registry‐based studies have investigated the impact of age at suicide bereavement per se, finding that risk of attempted and completed suicide is most pronounced in offspring who were bereaved by parental suicide in early childhood (Kuramoto et al., 2013; Niederkrotenthaler et al., 2012), increasing over decades of follow‐up (Kuramoto et al., 2013). Risk of suicide attempt in those who lost a parent to suicide in adolescence or young adulthood peaks within 2 years of the loss, then declining over follow‐up (Kuramoto et al., 2013). Findings from Dutch linkage data suggest that the elevated risk of suicide is more pronounced among offspring where the parent died by suicide prior to age 40 (Garssen et al., 2011), implying that younger age at bereavement is the critical exposure. Whist these studies highlight differing trajectories in the magnitude of suicide risk by age at parental suicide, and the likely impact of caregiver loss during a key developmental stage, our study focuses on a specific life stage for those bereaved at any age.

### Strengths and limitations

Strengths of this study include sampling of eligible individuals from the entire Danish population, with minimal loss to follow‐up and no issues of recall bias. Reliability and validity of linkage data has been evaluated as good with respect to suicide registration (Helweg‐Larsen, 2011; Tøllefsen et al., 2015), and hospital recording of self‐harm (Morthorst et al., 2016), including the precise timing of admissions for self‐harm in the Danish National Patient Register (Schmidt et al., 2015) and Psychiatric Central Research Register (Mors et al., 2011). This is important for a study investigating precise time periods. However, a reliance on hospital‐recorded self‐harm meant that we did not capture self‐harm recorded in primary care, nor community self‐harm without treatment‐seeking.

The SCCS approach is particularly well suited to rare outcomes and is a rigorous method of investigating temporal risk factors, maximizing power through all individuals serving as their own controls whilst also accounting for observed or unobserved confounders (Whitaker et al., 2006). Effect estimates are more precise than other observational designs (Farrington et al., 1996). Our restriction of the sample to offspring was pragmatic, given that risk may be kinship‐specific. It also dealt with the difficulties of accommodating wide variability in lead time before reaching age at parental death and in selecting appropriate comparison periods.

The limitations of our approach included that, to meet the conditions of the SCCS approach, we had to restrict the sample to offspring recorded with the outcome of self‐harm or suicide over our period of interest. We acknowledge that this excludes those who had died by suicide before our period of observation, those who did not present to services with self‐harm over the period of observation, and those who self‐harmed or died by suicide after this period. This sample is therefore not representative of all those losing a parent to suicide but provides important information about temporality of risk. Although our model did not account for any self‐harm in the period between bereavement and the start of the unexposed period (i.e., 15 years prior to the exposed period), this was addressed through the intra‐individual comparison. Our focus on secondary care data was intended to identify near‐lethal attempts, but may have under‐ascertained these events. Finally, despite evidence that suicide risk may be particularly pronounced after maternal suicide (Garssen et al., 2011), and in same‐gendered offspring to the deceased (Cheng et al., 2014), we did not investigate gender interactions due to lack of statistical power and risk of Type I error.

### Clinical and research implications

These findings suggest that bereaved people may benefit from greater support as they approach the age of a parent's suicide. Our findings support the practice of asking suicide‐bereaved individuals about age at parental suicide, identifying this as an anticipated period of increased risk and planning increased support. This is also an opportunity to reinforce that suicide is not inevitable after the suicide of a parent (Samata, 2022), with the absolute risk of suicide in offspring of suicide decedents estimated at less than 1% (Calderaro et al., 2022). This low estimate contrasts with the beliefs expressed by some suicide‐bereaved individuals (Pitman et al., 2017). Protective factors might be strengthened by inviting the bereaved person to consider ways to honor the deceased's memory (Pitman et al., 2018b) and safeguard the mental health of others affected by the loss (Pitman et al., 2017).

More research is required to understand the nature of distress experienced at this emotionally salient age, including whether risk of depression, anxiety, or substance use are also elevated. A grief reaction at this later stage is likely to resemble that of an acute grief response, including profound sadness and preoccupation with the deceased, anxiety related to the separation, and yearning for reunion (Shear & Skritskaya, 2012). There may also be a sense of renewed loss and/or loss of identity. Characterizing these difficulties will develop appropriate interventions. This, and an understanding of cognitive factors, will help describe the mechanisms of the association reported here. Work is needed to understand whether increased death thought accessibility, intensity of grief, reunion fantasy, or other cognitive factors drive the elevated suicidality observed at this point, informing intervention design. The concept of death thought accessibility (or mortality salience) describes the ability of bereaved people to defend themselves against the distress of loss by suppressing thoughts about death, particularly own vulnerability to death, by reducing self‐focused attention or trivializing the idea of an imminent vulnerability to death (Shear & Skritskaya, 2012). Suppression of death thoughts may be particularly challenging after very traumatic bereavements, such as those by suicide, but this has not been investigated. Factors that promote death thought accessibility after suicide loss might be modifiable risk factors for suicide. There is also a need for more evidence describing the phenomenon of post‐traumatic growth (Levi‐Belz et al., 2020; Armstrong & Shakespeare‐Finch, 2011) after suicide loss to understand its trajectory in different sub‐groups and the factors that promote it.

## CONCLUSIONS

Our population‐based study confirmed our hypothesis of an elevated risk of medically severe self‐harm and suicide among offspring upon attaining the age at which a parent died by suicide. Although mechanisms are not yet clear, suicide‐bereaved offspring may require increased support as they approach the age of parental death. More research is required to investigate the nature of cognitive factors contributing to this risk, including misconceptions about the heritability and inevitability of suicide, identification with the deceased, and yearning for reunion. This will inform appropriate interventions to reduce distress and risk of suicide.

## FUNDING INFORMATION

This work was supported by a grant from the American Foundation for Suicide Prevention (SRG‐0‐111‐17) to AP. GL is supported by a Sir Henry Dale Fellowship jointly funded by the Wellcome Trust and the Royal Society (grant number 223248/Z/21/Z). GL and AP are also supported by the National Institute for Health Research (NIHR) University College London Hospital (UCLH) Biomedical Research Centre (BRC). The funder was not involved in the design or conduct of the study; collection, management, analysis and interpretation of the data; preparation, review or approval of the manuscript; or decision to submit the manuscript for publication. Only AP, YL, KM, and AE had access to the raw data. The corresponding author had full access to all data used in the study, and final responsibility for the decision to submit for publication.

## CONFLICT OF INTEREST STATEMENT

The authors declare that they have no conflict of interest.

## ETHICS STATEMENT

The authors assert that all procedures contributing to this work comply with the ethical standards of the relevant national and institutional committees on human experimentation and with the Helsinki Declaration of 1975, as revised in 2013. The study was approved by the Danish Data Protection Agency (Capital Region of Denmark; P‐2020‐774) and the University College London Research Ethics Committee (approval number 14075/001). Informed consent was waived.

## Supporting information


Appendix S1.

## Data Availability

Danish registry data are available to researchers with appropriate affiliations on formal application to Statistics Denmark: https://www.dst.dk/en/TilSalg/Forskningsservice.
