# Supplementary material for: Risk of self‐harm and suicide on reaching the age at which a parent died by suicide or other causes: A Danish, population‐based self‐controlled case series study
Source: Suicide Life Threat Behav. 2024 Nov 19;55(1):e13135. doi: 10.1111/sltb.13135 (PMC11716354; doi:10.1111/sltb.13135)
Supplement: Supplementary file 1 — Appendix S1. [file SLTB-55-0-s001.docx]

## Supplementary Methods 1: Definitions of variables used to describe characteristics of samples

| **Category** | **Definition** | **Population register** |
| --- | --- | --- |
| **Causes of Death** |  |  |
| Suicide | ICD-8 codes E950-E959; ICD-10 codes X60-X84 and Y87.0 or where manner of death was recorded as ‘suicide’ | Register of Causes of Death [since 1980] |
| Other death | Any death recorded in the Causes of Death Registry except suicide | Register of Causes of Death [since 1980] |
| **Self-harm ^a^** | ICD-8 code E950-E959 or where ‘reason for contact’ was listed as self-harm  ICD-10 code X60-X84 or where ‘reason for contact’ was listed as self-harm. | Psychiatric Central Research Register [since 1969]  National Patient Register [since 1977] ^b^ |
| **Sociodemographic factors** |  |  |
| Age^ⴕ^ | Categorical variable, as 5-year age bands | Civil Registration System [since 1980] |
| Household income level^ⴕ^ | Quartiles: calculated as the total income within the household divided by the total number of adults living in the household, then categorised into quartiles based on national annual income averages i.e. relative to the whole Danish population. | Registry of Social Pension and Income [since 1980] |
| Marital status^ⴕ^ | Categorical variable: unmarried; married; widowed; divorced/separated.  Note that this variable did not include cohabitation status, as used in our main exposure definition, but was intended to capture the confounding effect of divorce. | Civil Registration System [since 1980] |
| Birth sex | Male/female | Civil Registration System [since 1980] |
| **Psychiatric Disorders^c^** |  |  |
| PTSD | ICD-8 code 309.81  ICD-10 code F43.1 | Psychiatric Central Research Register [since 1969] |
| Depression | ICD-8 codes 296.09, 296.2, 298.0, 300.4  ICD-10 codes F32.0-F32.2, F32.8-F32.9, F33.0-F33.2, F33.4-F33.9 | Psychiatric Central Research Register [since 1969] |
| Anxiety | ICD-8 codes 300.0, 300.2  ICD-10 codes F40, F41 | Psychiatric Central Research Register [since 1969] |
| Substance use | ICD-8 codes 291, 303, 304  ICD-10 codes F10-F19 (excluding F1x.0) **^d^** | Psychiatric Central Research Register [since 1969] |
| Severe mental illness (SMI)^e^ | ICD-8 codes 295, 296.89, 298.29-298.99, 299, 301.83, 296.1, 296.3, 298.1, 296.0 (excluding 296.09)  ICD-10 codes F20-F29, F30-F31, F32.3, F33.3 | Psychiatric Central Research Register [since 1969] |
| **Physical Disorders^f^** |  |  |
| Cardiovascular disease | ICD-8 codes 413, 410, 420, 425, 427.91, 427.93, 427.94, 427.09, 427.10, 427.11, 427.19, 427.99  ICD-10 codes I20-I22, I30, I42, I44, I46.0, I47.2, I48, I50 | National Patient Register [since 1977] ^b^ |
| COPD | ICD-8 codes 491-492, 518  ICD-10 codes J41-J44, J47 | National Patient Register [since 1977] ^b^ |
| Diabetes Mellitus | ICD-8 code 250  ICD-10 codes E105, E109, E111, E115, E119, E131, E135, E139, E141, E145, E149 | National Patient Register [since 1977] ^b^ |
| Hypertension | ICD-8 codes 400-404  ICD-10 codes I10-I13, I15 | National Patient Register [since 1977] ^b^ |

ICD-8= International Classification of Disease 8^th^ Revision (used in Denmark from 1969-1993)

ICD-10=International Classification of Disease 10^th^ Revision (used in Denmark from 1994-2016)

Note that ICD-9 was never implemented in Denmark, hence the transition from ICD-8 to ICD-10.

PTSD: post-traumatic stress disorder; COPD: chronic obstructive pulmonary disease.

^a^ Linkage to relatives was via the personal identification number in the Civil Registration System [since 1980]. For simplicity in defining the exposure risk periods, we replaced all births and deaths that occurred on 29 February among parents/study participants with 28 February.

^b^ The National Patient Register is sometimes termed the National Hospital Register.

^c^ Applies to codes/diagnoses recorded on inpatient psychiatric admissions.

^d^ Lowercase x denotes all possible values within the specified digit’s diagnostic category

^e^ SMI defined as psychotic disorders, manic episode, bipolar affective disorder, and depression with psychotic symptoms.

^f^ Applies to codes/diagnoses recorded on inpatient medical admissions.

^ⴕ^Time-varying covariate (seasonality was one of the four time-varying covariates used, operationalised as month at mid-point of each period, but is not shown in this table).

Supplementary Table 1: Risk of suicide attempt by specific periods over follow-up in a) suicide-bereaved individuals and b) other-bereaved individuals, censoring individuals on all second parental bereavements (IRRs) by risk period)

|  | **Event** | **PD** | **Unadjusted IRR**  **(95% CI)** | | **p-value** |  | | **Adjusted**†  **(95% CI)** | **p-value** |
| --- | --- | --- | --- | --- | --- | --- | --- | --- | --- |
| **Suicide-bereaved** |  |  |  |  | | |  |  |  |
| Years prior to exposed time period: |  |  |  | |  |  | |  |  |
| 16 - <13 years | 51 | 191,925 | 1.50 (0.97-2.31) | | 0.068 |  | | 0.63 (0.20-1.93) | 0.417 |
| 13 - <10 years | 42 | 191,767 | 1.24 (0.79-1.94) | | 0.360 |  | | 0.61 (0.26-1.43) | 0.254 |
| 10 - <7 years | 39 | 191,759 | 1.15 (0.72-1.82) | | 0.559 |  | | 0.73 (0.37-1.44) | 0.366 |
| 7 - <4 years | 49 | 191,749 | 1.44 (0.93-2.23) | | 0.102 |  | | 1.13 (0.69-1.86) | 0.619 |
| 4 - <1 year (reference period) | 34 | 191,750 | 1.00 [ref.] | | - |  | | 1.00 [ref.] | - |
| Exposed time period (Age of the deceased parent (2-year period) | 37 | 125,695 | 1.66 (1.04-2.64) | | 0.033 |  | | 2.03 (1.20-3.43) | 0.008 |
| After the age of the deceased parent: |  |  |  | |  |  | |  |  |
| 1 - <4 years | 26 | 159,842 | 0.89 (0.54-1.49) | | 0.671 |  | | 1.25 (0.65-2.42) | 0.499 |
| 4 - <7 years | 14 | 124,118 | 0.62 (0.33-1.16) | | 0.137 |  | | 1.14 (0.45-2.85) | 0.783 |
| 7 - <10 years | 5 | 90,737 | 0.30 (0.11-0.77) | | 0.013 |  | | 0.74 (0.20-2.76) | 0.656 |
| 10 - <13 years | 1 | 64,954 | 0.08 (0.01-0.60) | | 0.014 |  | | 0.26 (0.03-2.60) | 0.251 |
| 13 - <16 years ^ⴕⴕ^ | 0 | 50,933 | - | | 0.978 |  | | - | 0.977 |
|  |  |  |  | |  |  | |  |  |
| **Other-bereaved** |  |  |  | |  |  | |  |  |
| Prior to the age of the deceased parent: |  |  |  | |  |  | |  |  |
| 16 - <13 years | 232 | 730,419 | 1.84 (1.48-2.28) | | <0.001 |  | | 1.45 (0.80-2.61) | 0.217 |
| 13 - <10 years | 171 | 729,780 | 1.36 (1.08-1.71) | | 0.009 |  | | 1.12 (0.71-1.78) | 0.619 |
| 10 - <7 years | 167 | 729,776 | 1.33 (1.05-1.67) | | 0.017 |  | | 1.15 (0.81-1.64) | 0.426 |
| 7 - <4 years | 161 | 729,769 | 1.28 (1.01-1.61) | | 0.039 |  | | 1.18 (0.90-1.54) | 0.222 |
| 4 - <1 year (reference period) | 126 | 729,753 | 1.00 [ref.] | | - |  | | 1.00 [ref.] | - |
| Age of the deceased parent (2-year period) | 74 | 475,877 | 0.90 (0.68-1.20) | | 0.481 |  | | 0.98 (0.72-1.35) | 0.925 |
| After the age of the deceased parent: |  |  |  | |  |  | |  |  |
| 1 - <4 years | 85 | 585,631 | 0.86 (0.65-1.13) | | 0.280 |  | | 0.98 (0.69-1.38) | 0.897 |
| 4 - <7 years | 28 | 450,710 | 0.38 (0.25-0.58) | | <0.001 |  | | 0.49 (0.29-0.84) | 0.009 |
| 7 - <10 years | 18 | 341,584 | 0.33 (0.20-0.54) | | <0.001 |  | | 0.43 (0.22-0.86) | 0.017 |
| 10 - <13 years | 1 | 278,431 | 0.02 (0.00-0.17) | | <0.001 |  | | 0.04 (0.00-0.29) | 0.002 |
| 13 - <16 years ^ⴕⴕ^ | 0 | 239,123 | - | | 0.975 |  | | - | 0.978 |

**Legend:** CI: confidence interval; PDAR: person-days at risk

^†^Adjusted for age, marital status, household income level, and month (for seasonality).

^††^There were no events in this period due to the low number of individuals who had been bereaved at least 29 years before the end of data collection and to have survived until this period. As this was not the reference period, this did not bias estimates.

**Supplementary Table 2: Risk of self-harm or suicide at any point over follow-up in suicide-bereaved individuals and other-bereaved individuals (IRRs by risk period and aggregated intervening periods)**

|  | **Event** | **PDAR** | **Unadjusted IRR**  **(95% CI)** | | **p-value** |  | | **Adjusted**†  **(95% CI)** | **p-value** |
| --- | --- | --- | --- | --- | --- | --- | --- | --- | --- |
| **Suicide-bereaved** |  |  |  |  | | |  |  |  |
| 16 to 1 year before the loss | 228 | 1,030,184 | 1.00 [ref.] | | - |  | | 1.00 [ref.] | - |
| Age of deceased | 39 | 134,821 | 1.31 (0.93-1.83) | | 0.125 |  | | 1.93 (1.26-2.96) | 0.002 |
| 1 to 16 years after the loss | 49 | 530,741 | 0.42 (0.30-0.57) | | <0.001 |  | | 0.86 (0.51-1.43) | 0.555 |
|  |  |  |  |  | | |  |  |  |
| **Other-bereaved** |  |  |  |  | | |  |  |  |
| 16 to 1 year before the loss | 957 | 4,022,118 | 1.00 [ref.] | | - |  | | 1.00 [ref.] | - |
| Age of deceased | 85 | 524,615 | 0.68 (0.55-0.85) | | 0.001 |  | | 1.03 (0.80-1.33) | 0.833 |
| 1 to 16 years after the loss | 156 | 2,089,031 | 0.35 (0.30-0.42) | | <0.001 |  | | 0.71 (0.55-0.93) | 0.011 |

**Legend:** IRR: incidence rate ratio; CI: confidence interval; PDAR: person-days at risk.

†Adjusted for age, marital status, and household income level.
